# Mg_3_Pt_2_: Anionic Chains in a Eu_3_Ga_2_-Type Structure

**DOI:** 10.1021/acs.inorgchem.1c01995

**Published:** 2021-08-24

**Authors:** Laura Agnarelli, Yurii Prots, Ulrich Burkhardt, Marcus Schmidt, Primož Koželj, Andreas Leithe-Jasper, Yuri Grin

**Affiliations:** Max-Planck-Institut für Chemische Physik fester Stoffe, Dresden 01187, Germany

## Abstract

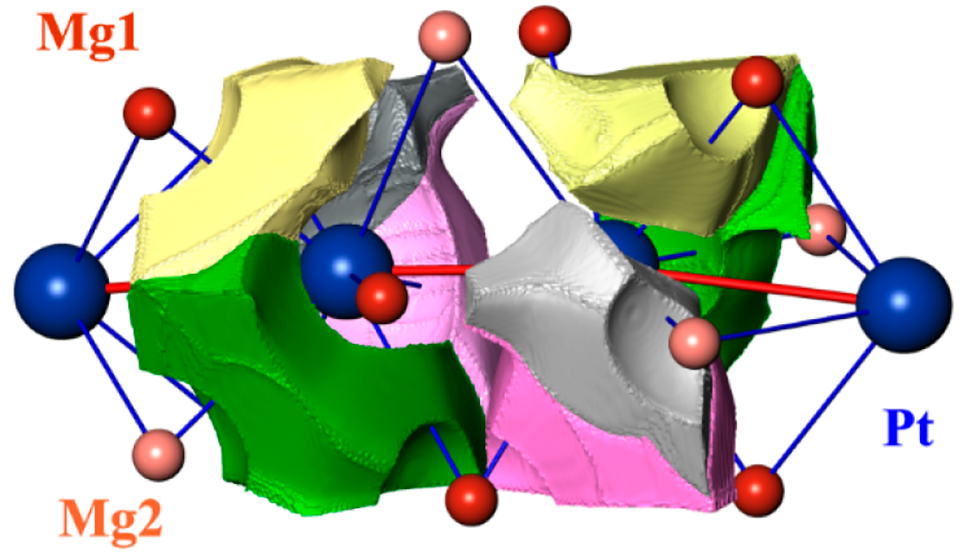

The binary phase Mg_3_Pt_2_ was prepared by direct reaction between the elements or by spark-plasma synthesis starting with MgH_2_ and PtCl_2_. The compound crystallizes in the monoclinic space group *C*2/*c* with *a* = 7.2096(3) Å, *b* = 7.1912(4) Å, *c* = 6.8977(3) Å, and β = 106.072(3)° and is isotypic to Eu_3_Ga_2_. Analysis of the electron density within the quantum theory of atoms in molecules shows a significant charge transfer from Mg to Pt in agreement with the electronegativity difference. Further study of the chemical bonding with the electron localizability approach reveals the formation of Pt chains stabilized by a complex system of multicenter interactions involving Mg and Pt species. The metallic character of Mg_3_Pt_2_ is confirmed by electronic structure calculations and physical measurements.

## Abstract 2

Mg_3_Pt_2_ is a new phase in the Mg−Pt system with a crystal structure built up by anionic Pt chains, which are stabilized by an interplay of multiatomic bonding interactions of Mg with Pt. Mg_3_Pt_2_ is a metallic diamagnet.

## 1 Introduction

The low density of Mg is considered to be a major advantage in manufacturing, where weight reduction is an issue of increasing importance.^1−3^ This particularly applies to the automotive sector and its needs for new structural materials.^4^ Light-weight Mg alloys with improved mechanical properties are therefore a scientific topic for applied and pure material sciences.^5−9^ Alloying elements like aluminum, copper, and zinc, together with other transition metals or rare-earth metals, are added to Mg to improve its strength, ductility, and creep and corrosion resistance.^10,11^ Besides the strong focus on structural applications, current research on Mg alloys as hydrogen-storage materials^12−14^ and their application as biomaterials should also be strongly emphasized.^15,16^ Pt-rich alkaline-earth-metal alloys currently attract interest as electrocatalytic materials for the oxygen reduction reaction in fuel cells.^17,18^ We also note that the use of Mg in extracting Pt from electronic or used-catalysator scrap has also been envisioned.^19^ Mg is a chemically reactive element, and it easily interacts with the alloying constituents, giving rise to the formation of intermetallic compounds.^20−23^ Besides their technological importance, exploring intermetallic compounds of Mg, their phase equilibria, and their structure–property relationships has been of general interest to the solid-state physics and chemistry community.^21,24−37^ The high affinity of Mg to Pt metals^38^ gives rise to a very variable structural chemistry,^39,40^ as has been elaborated for the Mg–Rh,^41−46^ Mg–Ir,^46−53^ and several Mg–Pd^54−63^ systems. For the Mg–Pt system, no phase diagram is reported in the literature; however, several intermetallic phases have already been described. The MgPt phase crystallizes with the FeSi structure type.^56^ Exposing Li_2_MgPt to hydrogen yields LiH and MgPt, which instead crystallizes with the CuAu structure type.^64^ A homogeneous solid solution forms around the Mg_6_Pt composition. The Mg_3_Pt compound crystallizes with the Na_3_As structure type.^65^ In another investigation, however, Mg_3_Pt was found to crystallize with the Cu_3_P type (a superstructure of Na_3_As).^66^ Mg_2_Pt, which adopts the tetragonal CuAl_2_ structure type, was synthesized later.^67^ Another two cubic phases, MgPt_3_ and MgPt_7_, were observed on the Pt-rich side of the Mg–Pt system.^68^ A material with composition Mg_2_Pt, for which the powder X-ray diffraction (PXRD) pattern was indexed with a C-centered monoclinic cell, was synthesized by the reaction between PtCl_2_ and either solid or solubilized MgH_2_ in organic solvents under mild conditions and a subsequent thermal treatment.^69^ A tentative structure solution was based on the structure of the high-pressure modification of titanium in a subcell with the space group *C*2/*m*, mentioning that a cell having a 4 times larger volume has the symmetry of the space group *C*2/*c*.^70^ In a thermodynamic study on the Mg–Pt system, the PXRD pattern of a phase with Mg_3_Pt_2_ composition was reported; however, not all of the reflections were indexed, and the structure of Mg_3_Pt_2_ remained unclear.^71^

Here we report a detailed study on the Mg_3_Pt_2_ phase, obtained either through a solid-state reaction between the elements or by use of the spark-plasma synthesis, its crystal structure, and chemical bonding.

## 2 Experimental Section

Samples with nominal composition Mg_3_Pt_2_ were prepared from elemental Mg (granules, Alfa Aesar, 99.98 wt %) and Pt (powder, Alfa Aesar, 99.9 wt %). Pt powder was added to the already induction-melted Mg granules on the bottom of a Ta tube. The Ta tube was then welded and heated up to 1080 °C in a high-frequency furnace for about 5 min. The melted product was ground and compacted into a pellet, and the latter was placed in an alumina crucible and sealed into a Ta tube for annealing at 800 °C for 2 weeks in a tube furnace. The reaction product is brittle and gray with a metallic appearance and stable in air. The complete sample preparation was performed in an argon-filled glovebox [MBraun, *p*(H_2_O/O_2_) < 0.1 ppm]. In an alternative way, the samples with nominal composition Mg_3_Pt_2_ were prepared from the powders PtCl_2_ (ChemPur, 99.9 wt %), MgH_2_ (in-house-synthesized^72^), and Pt (Alfa Aesar, 99.9 wt %) using the spark-plasma setup (SPS). Graphite dies with a diameter of 10 mm and a graphite lining were filled with approximately 1 g of the starting mixture and processed in an SPS-515 ET Sinter Lab apparatus (Fuji Electronic Industrial Co. Ltd., Tsurugashima, Japan) in an inert atmosphere. The reaction was performed in a vacuum (20 Pa) under uniaxial pressure (40 MPa), followed by slow cooling within the setup. The maximum temperature for the synthesis was constrained to 800 °C, reached within different rates, for a total preparation time of 130 min. After every experiment, the sample surface was cleaned from carbon and the product characterized by PXRD. The thermal behavior of the prepared materials was studied by differential scanning calorimetry (DSC; Netzsch DSC 404C Pegasus) using a closed Al_2_O_3_ crucible inside a sealed Ta ampule (see the Supporting Information). The chemical composition of the sample was determined by wavelength-dispersive X-ray spectroscopy (WDXS) performed on a Microprobe Cameca SX100 and scanning electron microscopy (SEM; Jeol JSM7800F) using Mg_2_Sn and elemental Pt as standards for Mg and Pt, respectively. The magnetic susceptibility of Mg_3_Pt_2_ was measured on a Quantum Design MPMS-XL 7T magnetometer in the temperature range from 1.8 to 300 K, and the electrical resistivity was measured on a Quantum Design PPMS in the temperature range 1.9–300 K (see the Supporting Information). PXRD experiments were performed with a Guinier–Huber Image Plate Camera G670 and Cu Kα_1_ radiation (λ = 1.54056 Å). Single crystals of Mg_3_Pt_2_ were selected from the crushed annealed samples. The splitters were glued to thin glass fibers and analyzed at room temperature using a Rigaku AFC7 diffraction system equipped with a Saturn 724+ CCD detector (Mo Kα radiation, λ = 0.71073 Å). The lattice parameters from the refinement of the PXRD and single-crystal X-ray diffraction data are in good agreement. Absorption correction was made by a multiscan procedure. All crystallographic calculations including the Rietveld refinement were performed with the program package *WinCSD*.^73^ Details of the data collection and further crystallographic information are listed in Table 1.

**Table 1:** Crystallographic Data for Mg_3_Pt_2_

composition	Mg_3_Pt_2_
space group	*C*2/*c*
Pearson symbol	*mS*20
formula units per unit cell, *Z*	4
unit cell parameters^a^	
*a* (Å)	7.2096(3)
*b* (Å)	7.1912(4)
*c* (Å)	6.8977(3)
β (deg)	106.072(3)
unit cell volume, *V* (Å^3^)	343.64(3)
calcd density, ρ (g cm^–3^)	8.940
cryst form	rectangular block
cryst size (μm^3^)	12 × 25 × 25
diffraction syst	Rigaku AFC7
detector	Saturn 724+ CCD
radiation, λ	Mo Kα, 0.71073 Å
scan, step/degree, *N*(images)	φ, 0.8, 450
2θ_max_	60°
range in *hkl*	–11 ≤ *h* ≤ 11, −5 ≤ *k* ≤ 10, −9 ≤ *l* ≤ 10
abs corrn	multiscan
abs coeff (mm^–1^)	81.27
*T*(max)/*T*(min)	0.381/0.140
*N*(*hkl*) measd	1257
*N*(*hkl*) unique	495
*R*(int)	0.043
*N*(*hkl*) obsd	436
observation criteria	*F*(*hkl*) ≥ 4σ[*F*(*hkl*)]
*R*_F_, *R*_W_	0.039, 0.041
residual density (e Å^–3^)	–0.79/1.12

a The lattice parameters were refined from individual peak positions extracted from the PXRD pattern recorded with Cu Kα_1_ radiation.

## 3 Calculation Procedures

The electronic structure calculation and bonding analysis were performed using the experimental values of the lattice parameters and atomic coordinates for Mg_3_Pt_2_ or employing the literature data for Eu_3_Ga_2_^74^ and K_3_Bi_2_.^75^ For Mg_3_Pt_2_, additional optimization of the atomic coordinates was performed to study the stability of the ELI-D maxima of the Pt–Pt bonds. The calculations were made with the all-electron, local orbital full–potential method (FPLO) within the local density approximation.^76^ The f states of europium were treated as valence states. Perdew–Wang parametrization was employed.^77^ Analysis of the chemical bonding in the position space^78,79^ for all three compounds was performed by means of the electron localizability approach. For this purpose, the electron localizability indicator (ELI) in its ELI-D representation^80,81^ and the electron density (ED) were calculated with a specialized module in the FPLO code.^82^ The topology of ELI-D and ED was evaluated by means of the program *DGrid*.^83^ The atomic charges from the ED and bond populations for bonding basins from ELI-D were obtained by the integration of ED and ELI-D, respectively, within the basins (space regions), bounded by zero-flux surfaces in the corresponding gradient field. This procedure follows the Quantum Theory of Atoms in Molecules (QTAIM).^84^ Combined analysis of the ED and ELI-D yields basic information for the description of the bonding situation in solids, in particular for intermetallic compounds.^85−89^

## 4 Results and Discussion

### 4.1 Compound Formation

**Scheme 1:**
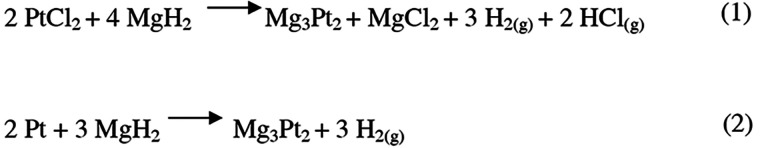
Alternative Routes for the Preparation of Mg_3_Pt_2_ Using the Spark-Plasma Synthesis with PtCl_2_ and MgH_2_ as Precursors (1) or Metallic Pt and MgH_2_ (2)

Using the direct reaction between the components, the existence of the Mg_3_Pt_2_ phase in the binary Mg–Pt system was confirmed. Its composition has been verified to be Mg_61(1)_Pt_39(1)_ from WDXS analysis. Once the exact composition of the phase was known, it was also synthesized following two alternative routes [reactions (1) and (2)] using the spark-plasma synthesis (Scheme 1).

Following reactions (1) and (2), Mg_3_Pt_2_ could not be obtained as a single phase because Mg_0.9_Pt_1.09_ was present as an impurity phase. From an experimental point of view, it was better to follow reaction (2) because the formation of MgCl_2_ from reaction (1) required its removal from the reaction product. The lattice parameters of the product obtained after reaction (2)—*a* = 7.2093(4) Å, *b* = 7.1949(9) Å, *c* = 6.8990(5) Å, and β = 106.078(8) deg—are within a few estimated standard deviations equal to the values of the product obtained from the elements (see below and Table 1). Thus, hydrogen uptake during the reaction can be excluded. From the DSC experiment, Mg_3_Pt_2_ decomposes congruently at 1214(1) °C (Figure S1).

### 4.2 Structure Determination

**Figure 1:**
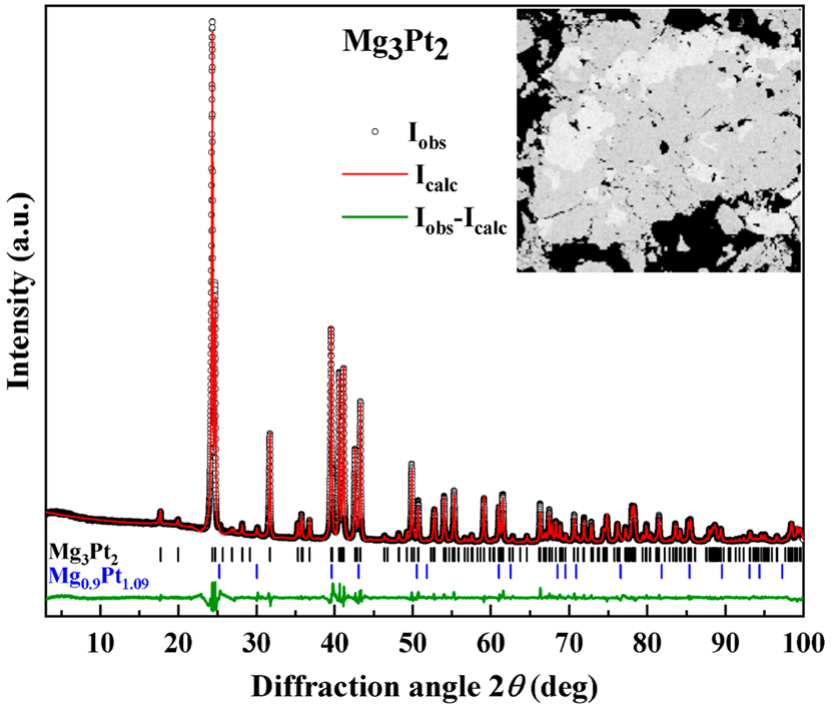
PXRD pattern of the Mg_3_Pt_2_ sample obtained by direct reaction of elemental metals, annealed at 800 °C for 2 weeks (Cu Kα_1_ radiation). The peak positions of the Mg_3_Pt_2_ and Mg_0.9_Pt_1.09_ phases are marked with black and blue sticks, respectively [refined phase content: main phase, 98.87(16)%; minor phase, 1.13(2)%]. Inset: Microstructure of the reaction product using a backscattering detector in the SEM. The dark-gray regions represent Mg_3_Pt_2_, and the light-gray fields belong to the Mg_0.9_Pt_1.09_ phase.

The PXRD pattern was indexed on the basis of a monoclinic unit cell [*a* = 7.2096(3) Å, *b* = 7.1912(4) Å, *c* = 6.8977(3) Å, and β = 106.072(3) deg]. All powder X-ray diffraction patterns showed small amounts [ca. 1.13(2)%] of the tetragonal phase Mg_0.9_Pt_1.09_ (isotypic to CuAu-type). This was also observed in the microstructure (Figure 1, inset).

Analysis of the diffraction intensities measured on a single crystal reveals systematic extinctions *h0l* for *h*, *l* = 2*n* and *h* + *l* = 2*n* for *hkl* reflections and indicates the possible space groups *C*2/*c* and *Cc*. The crystal structure was solved and refined in the space group *C*2/*c*. The Pt atom and one of the Mg atoms are situated on 8f sites, and the second kind of Mg atom occupies the 4e site. The obtained composition Mg_3_Pt_2_ well matches the WDXS analysis. The displacement of Pt atoms is described in the anisotropic approximation; together with the isotropic description of the Mg displacement, this yields the *R*_F_ value of 0.041, and the anisotropic description of Mg slightly reduces the *R* value to 0.039 (Tables 1 and S1). Because of the strong difference in the scattering power, the anisotropic description of Mg is not stable in the refinement. The anisotropy of the Pt atoms can be understood as the influence of the Pt–Pt bonding in the chain (cf. bonding analysis below): the ellipsoids are elongated along the Pt–Pt bond lines. The “flat” ellipsoids of Mg are squeezed along the Pt–Mg bond lines, reflecting the high ionicity of the bonding. The refined atomic coordinates and equivalent displacement parameters are listed in Table 2, and the anisotropic displacement parameters are listed in Table S1. The crystallographic data are deposited in the ICSD database with deposition number 2089451.

**Table 2:** Atomic Coordinates and Displacement Parameters of Mg_3_Pt_2_

atom	site	*x*/*a*	*y*/*b*	*c*/*z*	*U*_eq_^a^ (Å^2^)
Pt	8f	0.3750(1)	0.27358(9)	0.36175(9)	0.0099(2)
Mg1	4e	0	0.154(2)	0.25	0.014(2)
Mg2	8f	0.306(1)	0.0552(9)	0.023(1)	0.012(2)

a *U*_eq_ = 4/3[*U*_11_(a*)^2^*a*^2^ + ... 2*U*_23_(b*)(c*)*bc* cos(α)].

### 4.3 Structural Relationships

**Figure 2:**
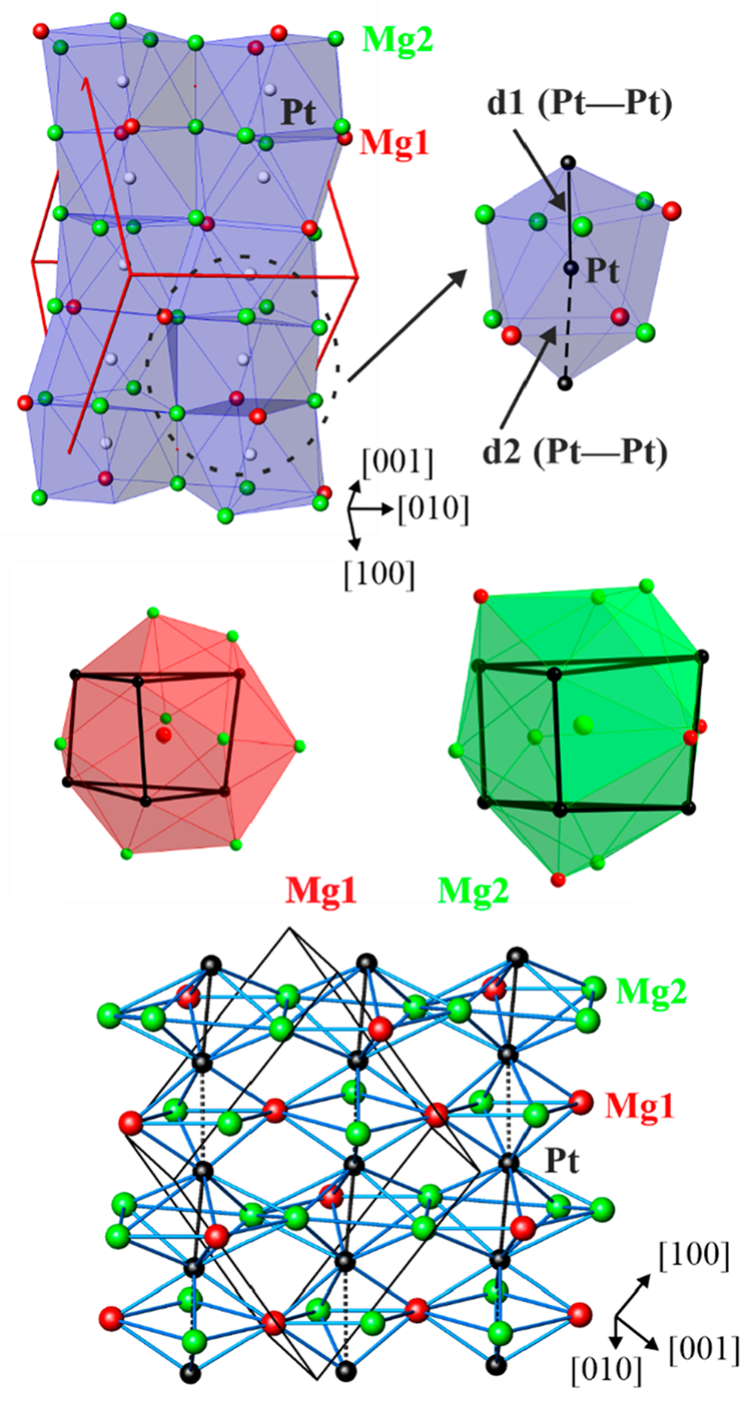
Crystal structures of Mg_3_Pt_2_. (top left) Undulating columns of distorted face-sharing [PtMg_9_] polyhedra. For simplicity, only two metal chains are drawn; the unit cell is highlighted in red. (top right) Coordination polyhedron of Pt. (middle) Coordination polyhedra of Mg1 and Mg2. (bottom) Interconnection of adjacent Pt chains through the sharing of vertices and edges of distorted octahedra Pt_2_(Mg1)_2_(Mg2)_2_ and distorted pentagonal bipyramids Pt_2_(Mg1)(Mg2)_4_.

Mg_3_Pt_2_ is isotypic to Eu_3_Ga_2_.^74^ The relevant interatomic distances in Mg_3_Pt_2_ are listed in Table 3. The striking feature of the crystal structure (Figure 2) is chains of Pt atoms, with alternating Pt–Pt distances. The shorter distance *d*_1_(Pt–Pt) = 2.68 Å is smaller than the Pt–Pt contact of 2.77 Å in face-centered-cubic (fcc) Pt,^90^ and the longer distance *d*_2_(Pt–Pt) amounts to 2.98 Å. A comparison between *d*_1_/*d*_2_ ratios for the Pt atoms in the Mg_3_Pt_2_ structure and the Ga atoms in the isotypic Eu_3_Ga_2_ structure^74^ shows that this ratio is smaller in Eu_3_Ga_2_ because of the much longer *d*_2_ Ga–Ga distances. Therefore, only Ga–Ga dumbbells are formed, while in Mg_3_Pt_2_, distorted Pt chains are present. Moreover, the chains in Mg_3_Pt_2_ are undulating with an angle slightly less than 180°. The Pt atom is located at the center of a distorted antiprism with coordination number (CN) = 11, in which nine Mg atoms form the two basal faces of the polyhedron, which is additionally capped by two Pt atoms. Such distorted [PtMg_9_] polyhedra are condensed by sharing faces and forming infinite undulating columns (Figure 2, top left). These columns can also be understood as being built from vertex-sharing distorted [Pt_2_(Mg1)_2_(Mg2)_2_] octahedra and pentagonal [Pt_2_Mg1(Mg2)_4_] bipyramids (Figure 2, bottom). The coordination polyhedra around the Mg1 and Mg2 atoms can be described as distorted trigonal prisms formed by Pt atoms capped by Mg with CNs of 14 and 15, respectively (Figure 2, middle).

**Table 3:** Interatomic Distances in the Crystal Structure of Mg_3_Pt_2_

atoms		distance (Å)	CN
Pt	Mg1	2.630(2)	
	Mg2	2.651(7)	
	Pt	2.677(1)	
	Mg2	2.712(7)	
	Mg2	2.715(6)	
	Mg1	2.736(3)	11
	Mg2	2.745(6)	
	Mg2	2.889(6)	
	Pt	2.984(1)	
	Mg1	3.046(9)	
	Mg2	3.244(7)	
Mg1	2 Pt	2.630(2)	
	2 Pt	2.736(3)	
	2 Mg2	2.897(9)	
	2 Pt	3.046(9)	
	2 Mg2	3.126(7)	14
	2 Mg2	3.362(9)	
	2 Mg2	3.392(11)	
Mg2	Pt	2.651(7)	
	Pt	2.712(7)	
	Pt	2.715(6)	
	Pt	2.745(6)	
	Pt	2.889(6)	
	Mg1	2.897(9)	
	Mg2	2.911(9)	
	Mg2	2.997(10)	
	Mg1	3.126(7)	15
	Pt	3.244(7)	
	Mg1	3.362(9)	
	Mg1	3.392(11)	
	2 Mg2	3.539(9)	
	Mg2	3.571(9)	

**Figure 3:**
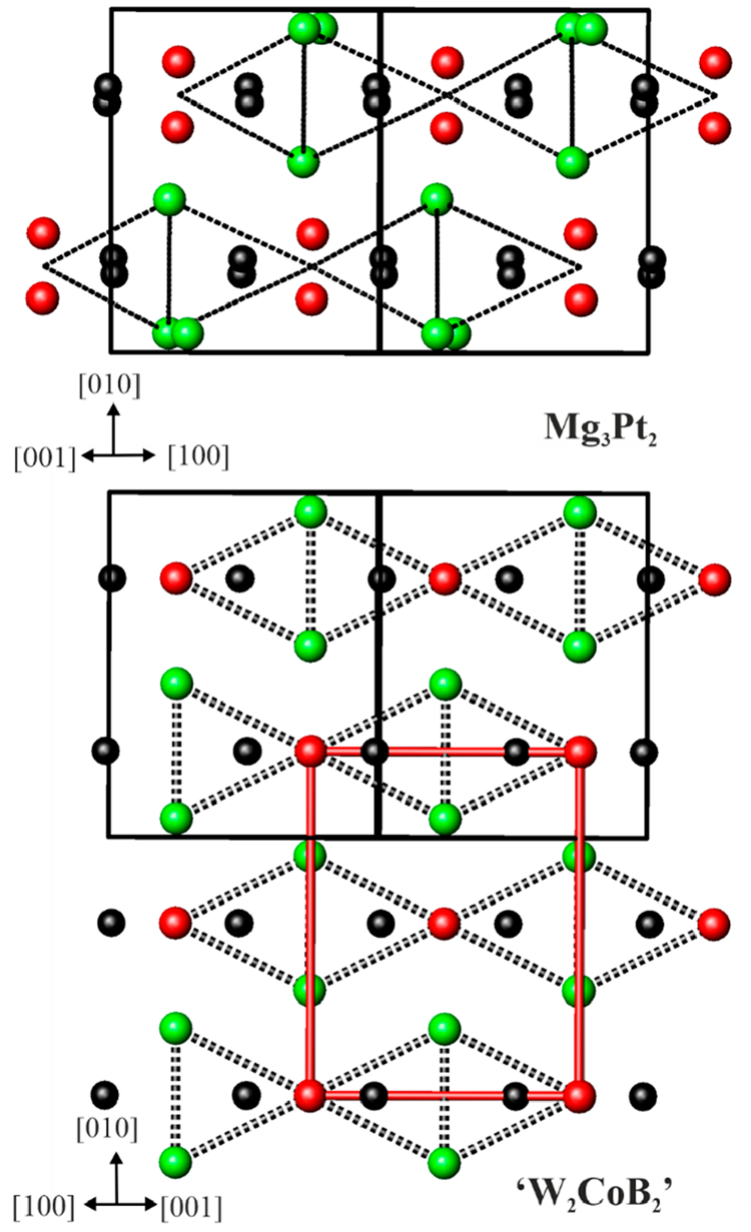
(top) Projection of the unit cell of Mg_3_Pt_2_ along the [101] direction. (bottom) Idealized atomic positions and the orthorhombic W_2_CoB_2_-type unit cell (red lines). Pt, Mg1, and Mg2 atoms are drawn in black, red, and green, respectively.

From a structural chemical perspective, Mg_3_Pt_2_ belongs to the Eu_3_Ga_2_ family. The latter can be structuraly related to the orthorhombic structures of W_2_CoB_2_^91^ and monoclinic Ca_2_SiIr_2_.^92^ If the atomic positions in Mg_3_Pt_2_ are shifted (Table S2 and Figure S3), an orthorhombic cell can be constructed (Figure 3, bottom). The atomic decoration of such a cell corresponds to a binary variant of the orthorhombic W_2_CoB_2_ type, where Mg atoms take the place of the two transition metals and the Pt atom replaces the B atom.

**Figure 4:**
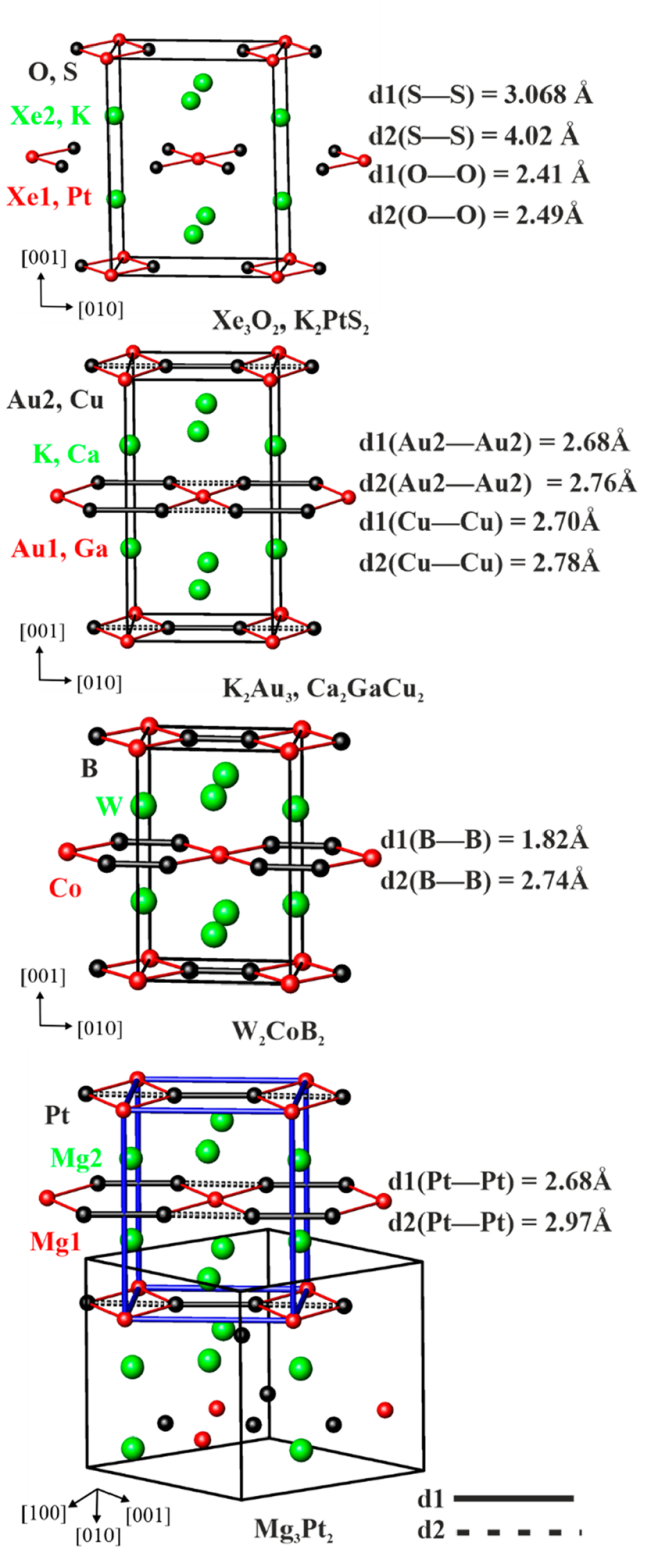
Comparison of orthorhombic W_2_CoB_2_ derivatives: *d*_1_ (black line), shorter distances between the atoms in the chain; *d*_2_ (dashed black line), longer distance between the atoms in the chain. The orthorhombic cell in Mg_3_Pt_2_ is highlighted in blue.

**Figure 5:**
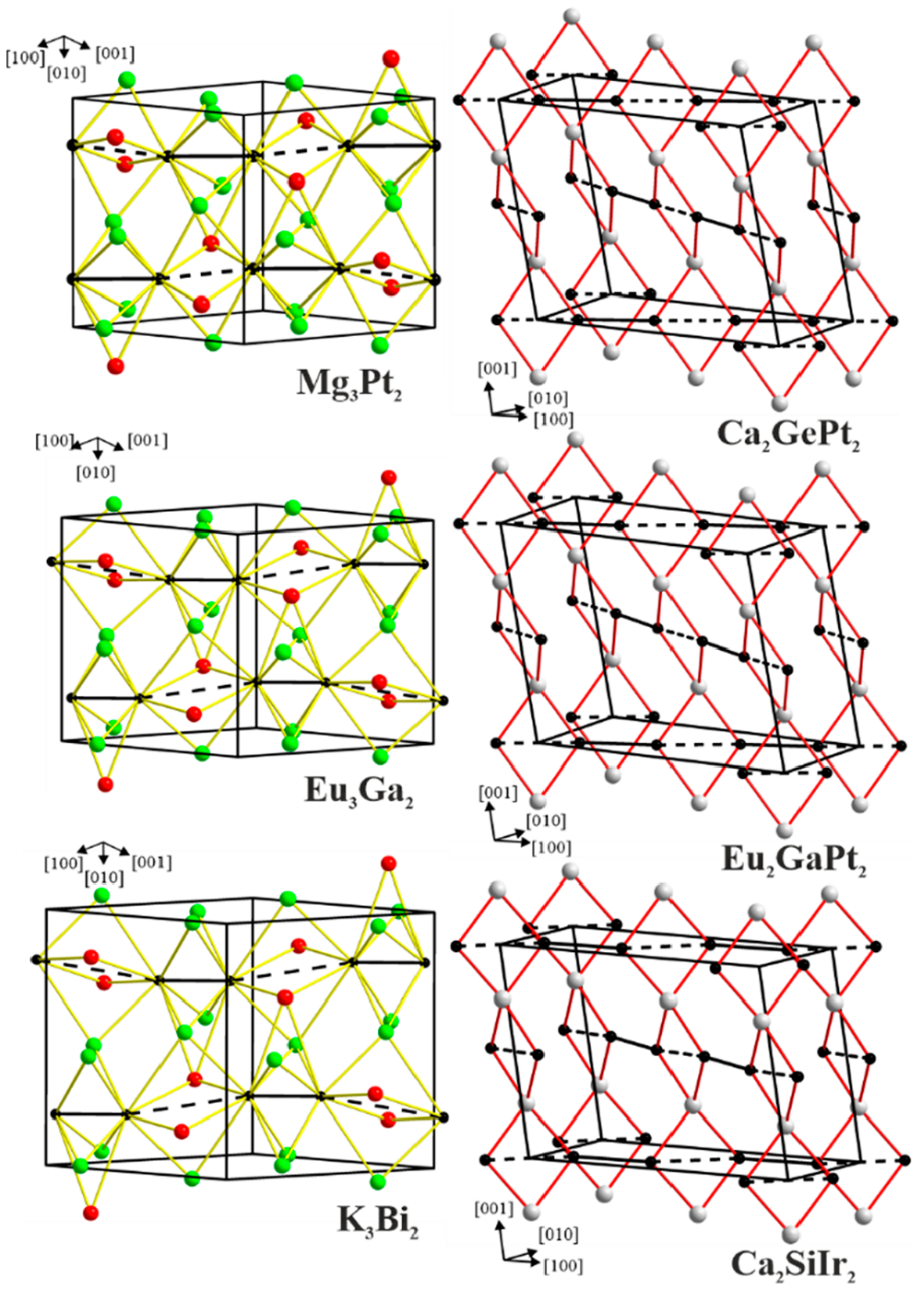
(left) Parallel metal chains in binary compounds of the Eu_3_Ga_2_ type with Mg1, Eu, and K cations acting as bridges between two parallel chains. (right) Almost perpendicular metal chains stacked along [001] in the compounds isotypic to Ca_2_SiIr_2_ bridged by Ga (Si and Ge) atoms.

Several compounds belonging to the W_2_CoB_2_ structural family are reported in the literature (Figure 4). They comprise different combinations of elements, bonding situations, and distance ratios *d*_1_/*d*_2_ in the chain. They can be described in terms of layered structures with atoms embedded between the layers. In W_2_CoB_2_,^91^ the dumbbells of B atoms [*d*_1_(B–B) = 1.82 Å] are aligned in the [010] direction and bridged by four Co atoms. The distance *d*_2_(B–B) = 2.74 Å is too long to expect an interaction between B atoms. This leads to the formation of layers {CoB_2_} separated by W atoms. A different situation is observed in K_2_Au_3_^93^ and Ca_2_GaCu_2_.^94,95^ The Au2 and Cu atoms form a continuous chain along [010] with alternating short and long distances, *d*_1_(Au2–Au2) = 2.68 Å, and the longest distance is *d*_2_(Au2–Au2) = 2.76 Å, respectively. In Ca_2_GaCu_2_, these two distances are 2.70 and 2.78 Å, respectively. In K_2_Au_3_, the distances *d*_1_ and *d*_2_ are both shorter than the shortest distances in elemental metallic Au (2.88 Å),^90^ pointing out the presence of Au chains in the structure. In Ca_2_GaCu_2_, the distances *d*_1_ and *d*_2_ are both longer than the normal Cu–Cu covalent bond distance of 2.55 Å,^90^ but they are still in a range in which one can expect Cu–Cu interaction. The layers of {Au1Au2_2_}and {GaCu_2_} are separated by the most electropositive elements K and Ca. Finally, in Xe_3_O_2_^96^ and K_2_PtS_2_,^97^ no bonding between S and O atoms is observed, and the structures contain XeO_4_ and PtS_4_ squares parallel to [010], which share O and S corners, respectively. In XeO_4_, Xe1 has an oxidation number of 4+, while the Xe2 atoms intercalated between the layer are unoxidized. In K_2_PtS_2_, the more electropositive K is intercalating between layers of PtS_4_ squares. Finally, in the idealized orthorhombic structure of Mg_3_Pt_2_, Pt chains with alternating distances are observed. From a structural point of view, the situation in Mg_3_Pt_2_ is similar to that observed in K_2_Au_3_, but the composition in K_2_Au_3_ is “inverted” compared to that of Mg_3_Pt_2_. Moreover, while in K_2_Au_3_ the layers of Au atoms only are separated by the electropositive K, in Mg_3_Pt_2_ the mixed {Mg1Pt2} layers are separated by Mg2 atoms. Anionic chains of the late transition-metal atoms are observed in a structurally related family of intermetallic compounds belonging to the Ca_2_SiIr_2_ type:^92^ Ca_2_GePt_2_ and Eu_2_GaPt_2_.^98,99^ While the space group is the same for the families (*C*2/*c*), in the latter ternary compounds, the metal chains are stacked along [001], being almost perpendicular to each other and bridged by Ga (Si and Ge) atoms (Figure 5, right). This is different from the almost parallel chains observed in Mg_3_Pt_2_, Eu_3_Ga_2_,^74^ and K_3_Bi_2_,^75^ where the Mg, Eu, and K atoms act as bridges between two parallel chains (Figure 5, left).

### 4.4 Chemical Bonding

**Figure 6:**
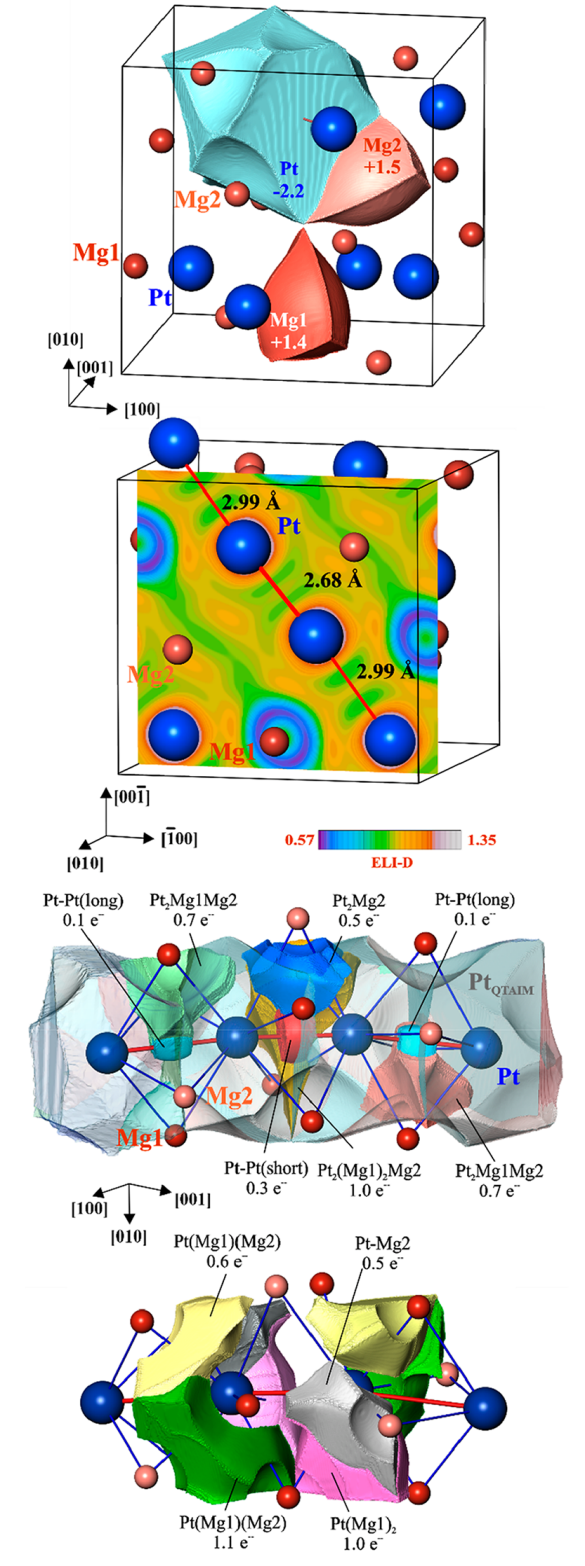
Chemical bonding in Mg_3_Pt_2_: (top) shapes of the QTAIM atoms and their charges; (upper middle panel) ELI-D distribution in the plane of the Pt chain; (lower middle panel) ELI-D basin of the bonding interactions in the Pt chain involving two Pt atoms (color code) and the QTAIM Pt atoms (transparent); (bottom) ELI-D basin of the bonding interaction in the Pt chain involving one Pt atom.

**Figure 7:**
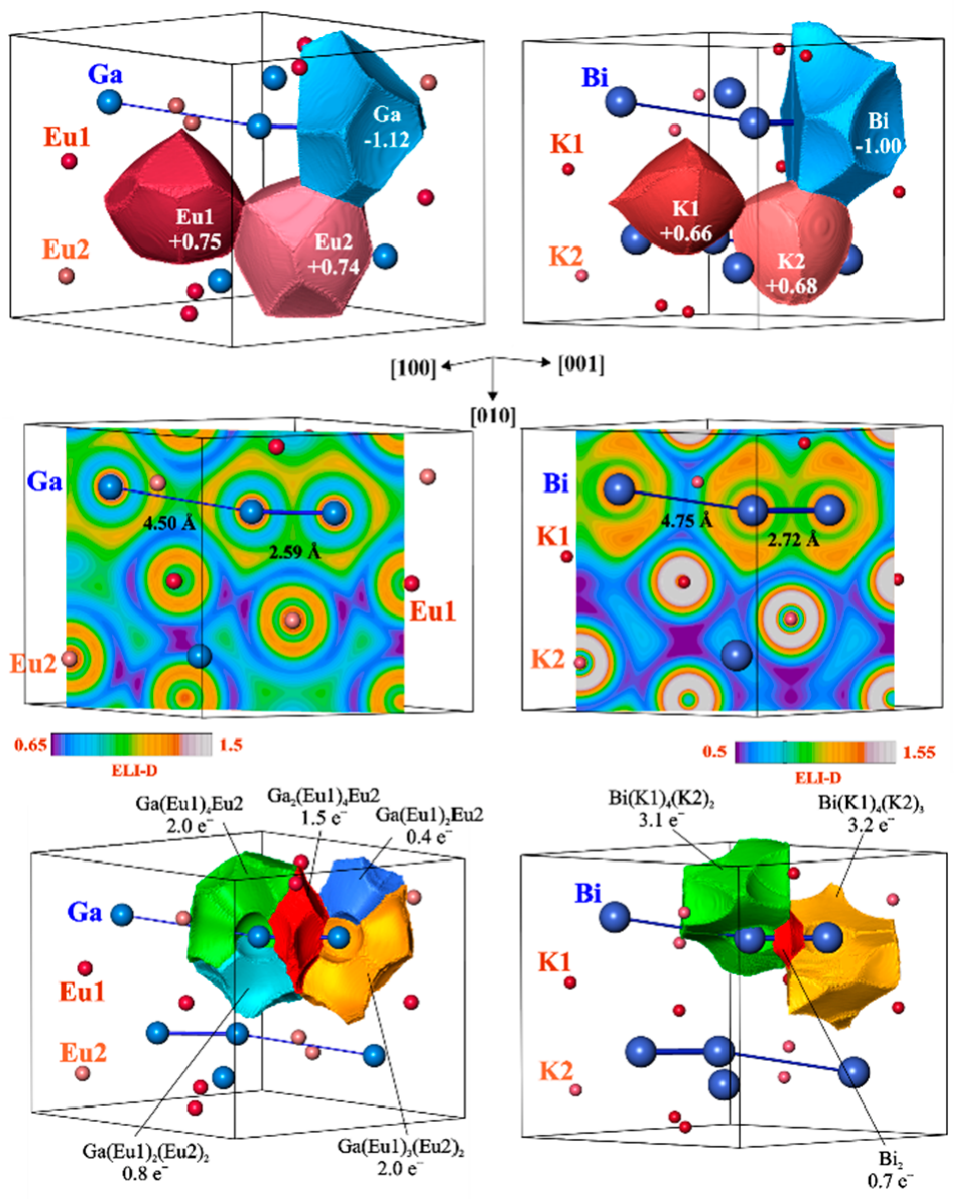
Chemical bonding in Eu_3_Ga_2_ (left) and K_3_Bi_2_ (right): (top) QTAIM atoms and their effective charges; (middle) ELI-D distribution in the plane of the Ga or Bi chains; (bottom) ELI-D basins of bonding interactions involving Ga or Bi atoms.

From the crystallographic point of view, the crystal structure of Mg_3_Pt_2_ can be described in different ways, depending on the structural arrangement that is used for comparison (see above). In order to gain more insight into which of this description has its roots in the atomic interactions, analysis of the chemical bonding is performed by applying the electron localizability approach, a quantum-chemical technique operating in the position space. Topological analysis of the electronic density following the QTAIM theory yields the shapes of the constituting atoms (Figure 6, top panel). Integration of the ED within these atomic basins results in the effective charges of −2.2 for Pt and +1.4 and +1.5 for the two symmetry-independent Mg species Mg1 and Mg2, respectively. The essential charge transfer is in agreement with the electronegativity difference between Pt and Mg. The shapes of the Mg cations are similar to the Be species in Be_21_Pt_5_^100^ and Be_5_Pt^101^ and show clear deviation from a sphere (including mainly the inner shells), characteristic for the (formally divalent) cations, e.g., in intermetallic clathrates.^102,103^ The markedly larger shape of the Pt anion contains plane faces toward the Pt neighbors and concave faces toward the cations, indicating interactions of different polarities. Analysis of the literature information reveals only five binary compounds belonging to the Eu_3_Ga_2_ type.^74^ Besides the prototype and Mg_3_Pt_2_, this family includes three bismuthides of alkaline metals, K_3_Bi_2_, Rb_3_Bi_2_, and Cs_3_Bi_2_.^75^ QTAIM analysis of the ED for Eu_3_Ga_2_ and K_3_Bi_2_ confirms the important role of charge transfer for stabilization of the atomic arrangement of this type (Figure 7, top panel). Nevertheless, the absolute charge values are noticeably smaller in comparison with Mg_3_Pt_2_ (cf. −1.12 for Ga and −1.0 for Bi with the approximately double negative charge of Pt).

Distribution of the ELI in Mg_3_Pt_2_ reveals maxima in the vicinity of the Pt chains with alternating Pt–Pt distances (Figure 6, upper middle panel). They can be classified in three groups. The one group visualizes the Pt–Pt interactions (red and light blue in the lower middle panel in Figure 6), confirming the formation of a Pt chain. These basins remain also after optimization of the structure (cf. Calculation Procedures). The populations of these basins are rather low (0.1–0.3 e^–^), indicating that they do not seem to play a key role. This function carries multiatomic interactions of the second and third kinds. The basins of the second kind of attractors have contact with the core basins of two neighboring Pt atoms and other Mg species. They visualize the multiatomic interactions “bridging” the Pt–Pt bonds (Figure 6, lower middle panel). The attractors of the third group reveal the three- and two-atomic interactions with Mg involving one Pt atom only (Figure 6, bottom panel). The basin populations of the second and third groups of attractors (0.5–1.1 e^–^) are essentially larger than that of the Pt–Pt bonds. Thus, the Pt chain in Mg_3_Pt_2_ is stabilized by a complex system of two-, three-, and four-atomic bonds involving Pt species from the chains. The intersection of these bond basins with the QTAIM atomic basins of Pt clearly shows that the latter contribute the majority of the populations; i.e., all interactions involving Pt and Mg are strongly polar. The presence of numerous multiatomic interactions in the structure correlate with the calculated electronic density of states (DOS; Figure S2), which is mainly formed by the Pt *d* states with small contributions of the Mg *s* states and has a low but nonnegligible DOS at the Fermi level. The experimentally found metallic behavior of Mg_3_Pt_2_ is also in line with the DOS (Figure S4).

Topological analysis of ELI-D for Eu_3_Ga_2_ yields, despite the same structure type, an essentially different bonding picture (Figure 7, left middle and bottom panels). A two-atomic Ga–Ga interaction is observed only for the shorter Ga–Ga contact. Its basin has a population of 1.5 e^–^, being much larger in comparison to the Pt–Pt bonds in Mg_3_Pt_2_. Besides the major contributions of two Ga atoms to the basin, also a small portion comes from the five bridging Eu species. The remaining basins (four for each Ga) involve each contribution from one Eu and several Ga atoms and visualize lone-pair-like multiatomic bonding. In total, chemical bonding in Eu_3_Ga_2_ reveals, within the “chain”, the formation of separated Ga_2_ dumbbells with bridged Ga–Ga bonds. The dumbbells are separated by multicenter lone-pair-like interactions. The next representative of the Eu_3_Ga_2_ type, K_3_Bi_2_, is also characterized by the formation of Bi2 dumbbells (Figure 7, right middle panel). In this case, the Bi–Bi interaction is clearly a two-atomic one: only two-atomic basins of Bi contribute to the bonding basin (population of 0.7 e^–^; Figure 7, right bottom panel).

Each Bi species has on the bond-opposite side two lone-pair-like basins visualizing the polar seven- and eight-atomic interactions of Bi with its K ligands. The populations of these basins (3.1 and 3.2 e^–^) are the largest in the series of the three Eu_3_Ga_2_-type representatives studied. In summary, the common bonding feature of the Eu_3_Ga_2_-type representatives is the polar interaction between different components. The polarity increases with increasing electronegativity difference between the constituents. The main structural feature, the formation of a homoatomic anionic chainlike arrangement, depends on the number of available electrons per formula unit (VE). For the larger numbers (VE of 13 for K_3_Bi_2_ [VEC(Bi) = 6.5] and VE of 12 for Eu_3_Ga_2_ [VEC(Ga) = 6]), the formal chain splits into separate dumbbells. The total number of electrons in the bonding basins around the dumbbells are 13.3 and 11.9, respectively, being close to the VE values. For lower numbers of electrons available (VE = 8 in Mg_3_Pt_2_ [VEC(Pt) = 4]), the chain arrangement with alternating distances is stabilized. The total number of electrons in the bonding basins (per two Pt atoms) is 10.7, revealing participation of the penultimate-shell electrons of Pt in the bonding.

## 5 Conclusions

The binary compound Mg_3_Pt_2_ was obtained either by direct reaction of elemental metals or by spark-plasma treatment of mixtures of PtCl_2_ and MgH_2_. Mg_3_Pt_2_ is isotypic with Eu_3_Ga_2_ (and A_3_Bi_2_, where A = K, Rb, and Cs) but differs clearly from the latter compounds from the chemical bonding point of view. While, because of the sufficient number of valence electrons available, the minority atoms in Eu_3_Ga_2_ and A_3_Bi_2_ form isolated dumbbells arranged along a line with a huge difference between the short and long distances within such a “chain”, the Pt atoms in Mg_3_Pt_2_ form almost linear chains with only moderate alternating Pt–Pt distances in the chain. Analysis of the chemical bonding reveals multiatomic interactions stabilizing the Pt–Pt chain, while the dumbbells in Eu_3_Ga_2_ and A_3_Bi_2_ are separated within the line by “lone pairs” (strongly polar multiatomic interactions). The metallic and diamagnetic character of Mg_3_Pt_2_ is confirmed by physical property measurements.

## Supporting Information Available

Anisotropic displacement parameters, thermal analysis, calculated DOS, idealized coordinates for Mg_3_Pt_2_, and physical properties (PDF)

## Accession Codes

CCDC 2089451 contains the supplementary crystallographic data for this paper. These data can be obtained free of charge via www.ccdc.cam.ac.uk/data_request/cif, or by emailing data_request@ccdc.cam.ac.uk, or by contacting The Cambridge Crystallographic Data Centre, 12 Union Road, Cambridge CB2 1EZ, UK; fax: +44 1223 336033.
