# Supplementary material for: Mg3Pt2: Anionic Chains in a Eu3Ga2-Type Structure
Source: Inorg Chem. 2021 Aug 24;60(17):13681–90. doi: 10.1021/acs.inorgchem.1c01995 (PMC8424623; doi:10.1021/acs.inorgchem.1c01995)
Supplement: Supplementary file 1 — ic1c01995_si_001.pdf [file ic1c01995_si_001.pdf]

Supporting Information

Mg_3Pt_2: anionic chains in a Eu_3Ga_2-type structure

Laura Agnarelli[1], Yurii Prots[1], Ulrich Burkhardt[1], Marcus Schmidt[1], Primož Koželj[1], Andreas Leithe-Jasper[1], Yuri Grin[1]*

[1] Max-Planck- Institut für Chemische Physik fester Stoffe, Nöthnitzer Straße 40, 01187 Dresden, Germany

*email: Andreas.Leithe-Jasper@cpfs.mpg.de

Table of Contents

Table S1 ……………………………………………………………………………………S2
S1. Thermal Analysis……………………………………………………………………S3
S2. Calculated electronic density of states……………………………………………S4
Table S2……………………………………………………………………………………S5
S3. Idealized coordinates for Mg_3Pt_2 in the W_2CoB_2 setting………………………S6
Table S3……………………………………………………………………………………S7
S4. Resistivity measurements……………………………………………………………S8
S5. Magnetoresistance measurements…………………………………………………S9
S6. Magnetic properties……………………………………………………………………S10

Table S1. Anisotropic displacement parameters ($Å^2$) for Mg_3Pt_2

Atom	U_{11}	U_{22}	U_{33}	U_{12}	U_{13}	U_{23}
Pt	0.0171(4)	0.0085(3)	0.0055(3)	0.0008(2)	0.0058(2)	-0.0012(2)
Mg1	0.020(4)	0.021(5)	0.005(4)	0	0.007(3)	0
Mg2	0.022(3)	0.006(3)	0.011(3)	-0.004(2)	0.010(3)	-0.002(2)

S1. Thermal Analysis

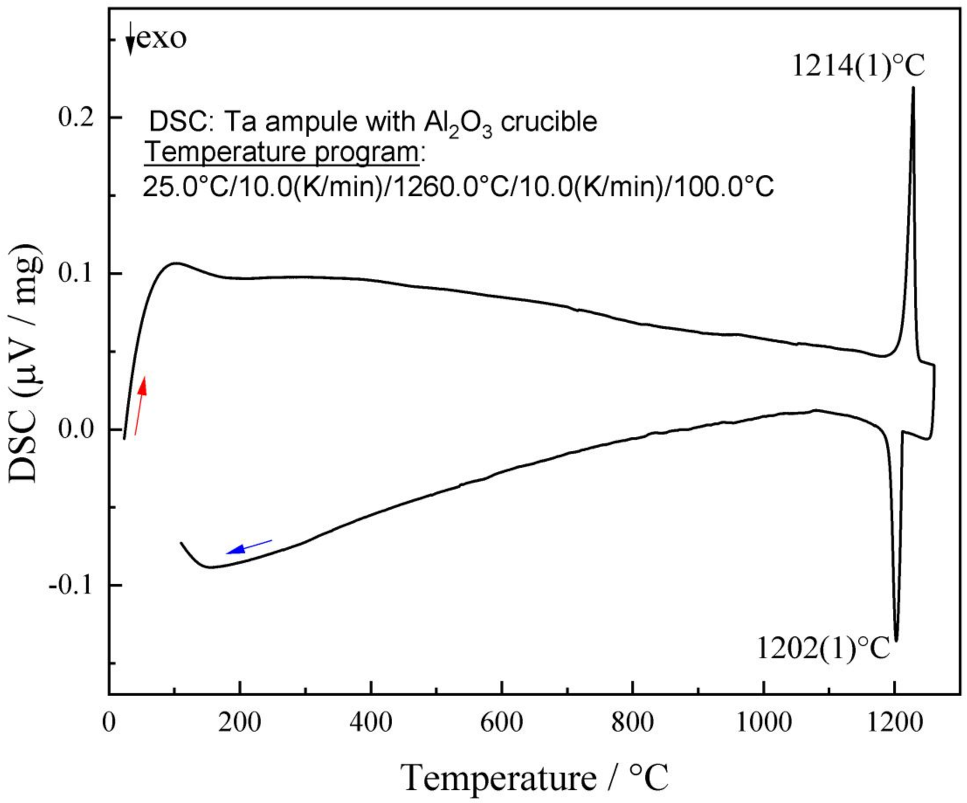

Figure S1. Differential scanning calorimeter (DSC) of Mg_3Pt_2.

The thermal behavior of the Mg_3Pt_2 phase was carried out using a Netzsch DSC 404C Pegasus, in flowing argon atmosphere (Ar 99.999% 100 ml/min with subsequent drying and oxygen post-purification via Big Oxygen Trap by Trigon Technologies) and using a thermocouple type S. The analyzed sample mass was 83 mg and the rate of heating and cooling 10.0 K/min. The graphic in Figure S1 shows that Mg_3Pt_2 phase formed congruently at 1214(1)˚C (onset temperature).

S2. Calculated electronic density of states

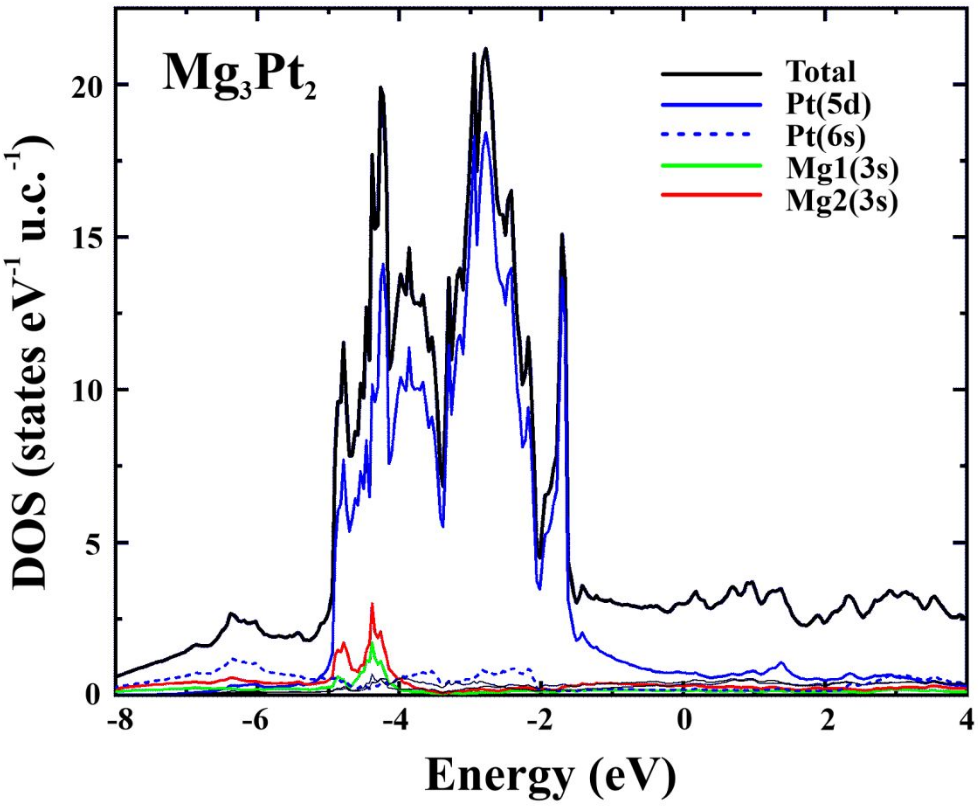

Figure S2. Calculated electronic density of states (DOS) for Mg_3Pt_2.

Table S2. Idealized coordinates for Mg_3Pt_2 in the W_2CoB_2 setting.

Atom	Site	*x/a*	*y/b*	*c/z*
Pt	8*f*	0.3750(1)	1/4	0.3617(1)
Mg1	4*e*	1/2	1/4	1/4
Mg2	8*f*	1/4	0.0552(1)	1/2

S3. Idealized coordinates for Mg_3Pt_2 in the W_2CoB_2 setting

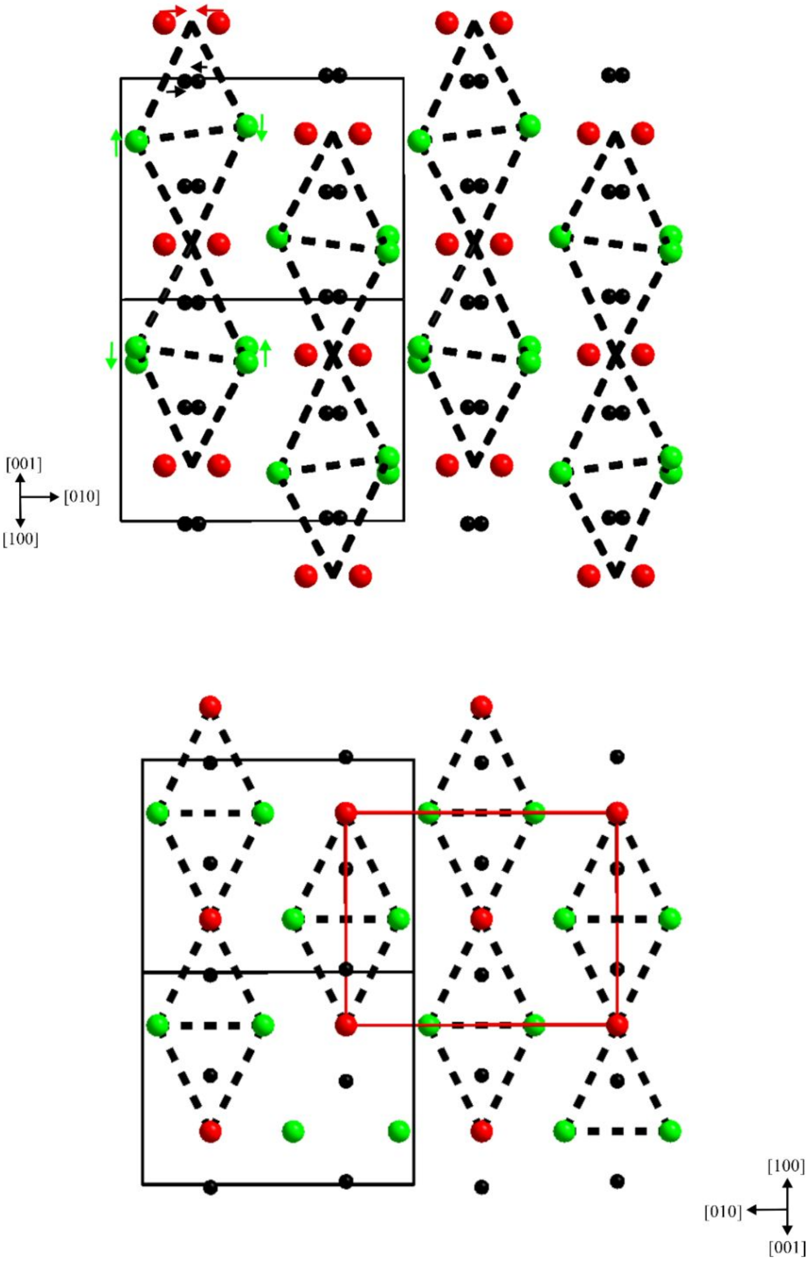

Figure S3. (Top) Projection of the monoclinic unit cell of Mg_3Pt_2 along [101]. With red, green and black arrows are indicated the shifts of Mg1, Mg2 and Pt atoms respectively to obtain the idealized orthorhombic arrangement. (Bottom) Idealized atomic positions and orthorhombic sub-cell of W_2CoB_2 type (red).

Table S3. Comparison of Electron counting and distances in the metal chains in the Eu_3Ga_2 family compounds

	d_1/d_2	r(M1)	r(M2)	VE
Mg_3Pt_2	1.11(1)	1.60	1.39	8
Eu_3Ga_2	1.74(1)	1.99	1.53	12
Cs_3Bi_2	1.72(1)	2.72	1.82	13
Rb_3Bi_2	1.75(1)	2.50	1.82	13
K_3Bi_2	1.75(1)	2.35	1.82	13

M1: electropositive elements

M2: Pt, Ga, Bi

S4. Resistivity measurements

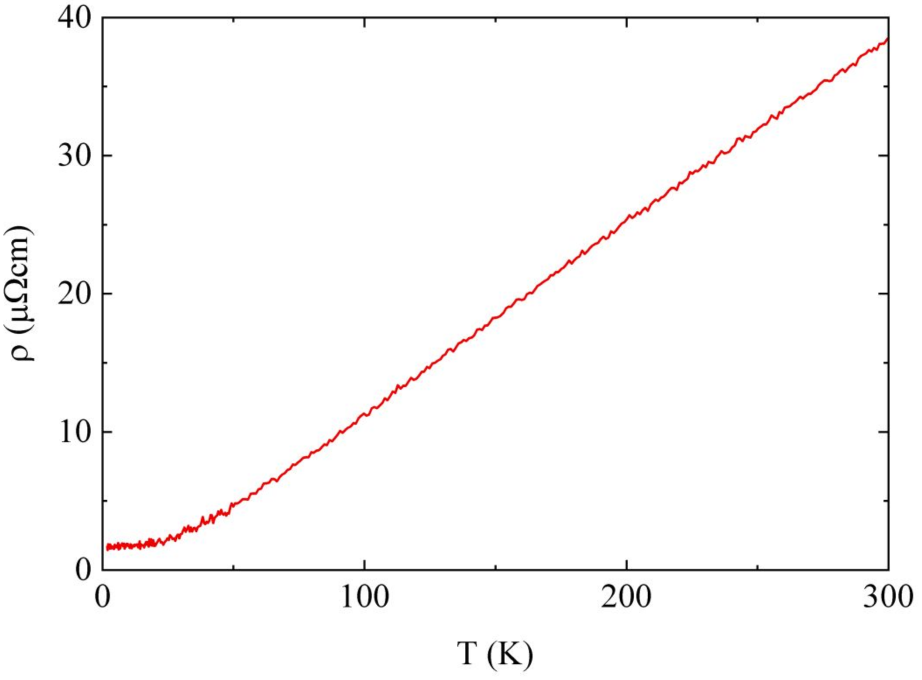

Figure S4. Resistivity measurements on Mg_3Pt_2 in the temperature range 1.9 K to 300 K.

The electrical resistivity of Mg_3Pt_2 was measured in the temperature range 1.9 K to 300 K. Note that the sample used for electrical resistivity measurements is not a perfect single crystal but an agglomerate of polycrystalline grains due to limitations of the sample synthesis method.

The temperature dependence in zero magnetic field (Figure S4) displays typical metallic behavior and a residual resistivity of 1.7 μΩcm. The metallic behavior corroborates the non-zero density of states at the Fermi level as calculated by density functional theory with the FPLO code, see Figure S2. To complement the zero-magnetic-field measurement of resistivity, the dependence of

resistivity on magnetic field was collected at two fixed temperatures, 1.9 K and 100 K (Figure S5, main panel). These magnetoresistance measurements are plotted as $\Delta\rho/\rho=(\rho(B)-\rho(0))/\rho(0)$ so that they represent change compared to zero-field resistivity at that particular temperature. At both temperatures the magnetoresistance linearly depends on magnetic field and reach at 1.9 K and 100 K values of 175% or 25%, respectively, in our maximum magnetic field of 90 kOe.

S5. Magnetoresistance

To determine the temperature range for sizeable magnetoresistance effects, a resistivity versus temperature measurement was also performed in a magnetic field of 90 kOe, (inset of Figure S5). Consistent with the resistivity vs. magnetic field measurements, the 90 kOe measurement displays at low temperature significantly increased values compared to the resistivity in zero magnetic field. The difference between the measurement with and without a magnetic field decreases with temperature but remains visible up to room temperature.

The authors cannot at this moment provide a satisfactory explanation for these magnetoresistance values which are high for metallic systems but we speculate that probably the shape of the (anisotropic) Fermi surface is such that open orbits are possible in one or more directions leading to an enlarged magnetoresistance. Likely synthesis of higher quality crystals and/or an in depth DFT study would be needed.

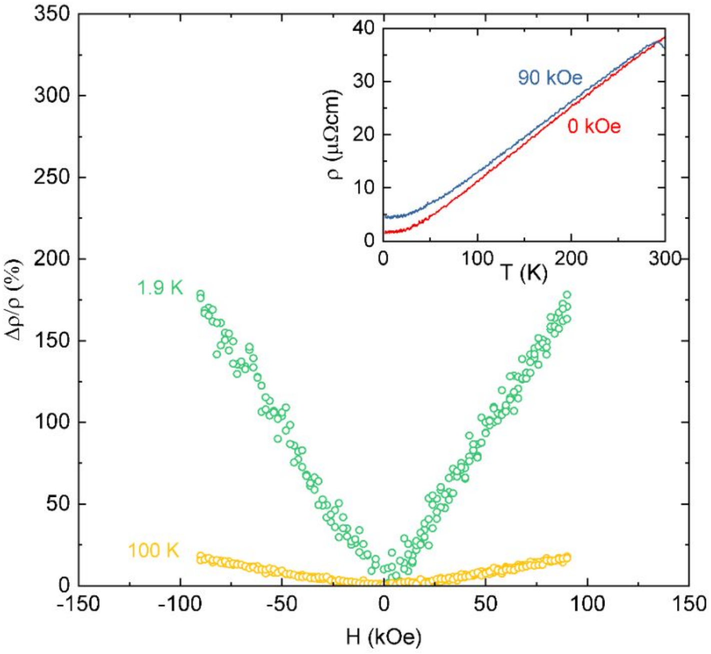

Figure S5. Magnetoresistance in Mg_3Pt_2. Main panel: The magnetoresistance of Mg_3Pt_2 goes up to 175% or 25% for 1.9 K and 300 K, respectively, in our maximum magnetic field of 90 kOe. Inset: Resistivity versus temperature for zero magnetic field and 90 kOe shows that magnetoresistance decreases towards room temperature but still remains non-negligible.

S6. Magnetic properties

The magnetic susceptibility of Mg_3Pt_2 was measured in the temperature range from 1.8 K to 300 K, see Figure S6. The measured signals were negative at higher temperature corresponding to an overall diamagnetism of the sample but exhibited paramagnetic tails at low temperature. The data displayed in Figure S6 is after a simple Honda-Wilson-like correction[1-2] which was used to compensate for a slight shift of the curves at high temperature due to ferromagnetic impurities in the sample. The required shift corresponds to 0.0001 wt.% of iron, which is a quantity which could be reasonably attributed to either impurities in the chemicals or contamination during sample handling. The overall diamagnetism of the sample can be understood as a sum of two contributions. The first is the Pauli paramagnetism of the conduction electrons (Figure S5) and the related discussion showing metallic behavior of Mg_3Pt_2. The second contribution is the diamagnetism of the closed shells, which in our case can be estimated to amount to a molar susceptibility cca $0.08 \cdot 10^{-3}$ emu/mol [3], meaning that the Pauli paramagnetism and the diamagnetism of closed shells almost fully cancel out giving an overall susceptibility of $0.02 \cdot 10^{-3}$ emu/mol.

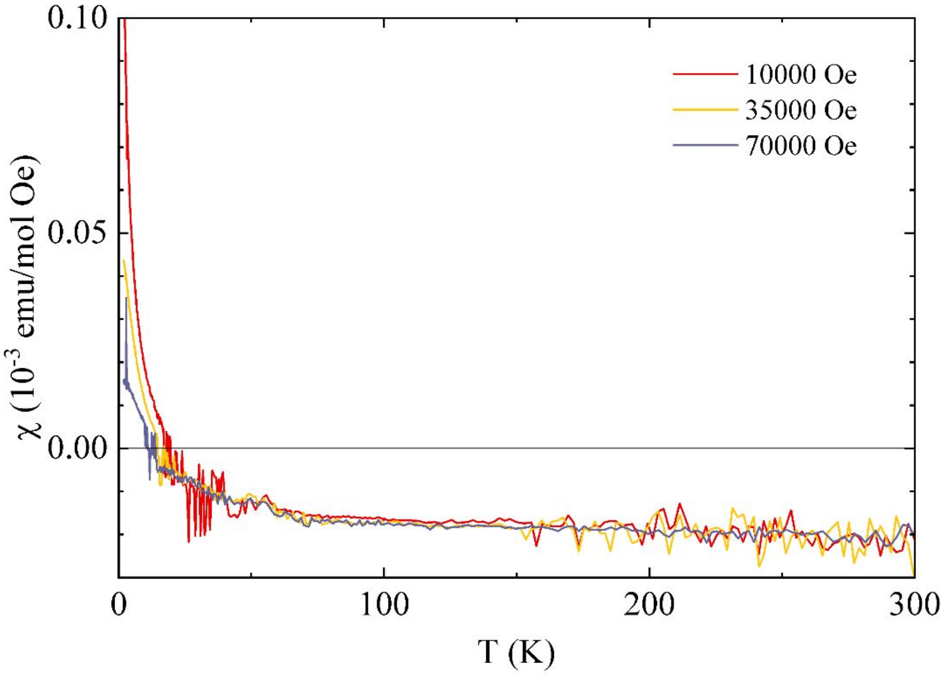

Figure S6. Molar magnetic susceptibility of Mg_3Pt_2 normalized per mole of formula unit.

References

1. Honda, K., Die thermomagnetischen Eigenschaften der Elemente. *Annalen der Physik* **1910,** *337* (10), 1027-1063.

2. Owen, M., Magnetochemische Untersuchungen. Die thermomagnetischen Eigenschaften der Elemente. II. *Annalen der Physik* **1912,** *342* (4), 657-699.

3. Bain, G. A.; Berry, J. F., Diamagnetic Corrections and Pascal's Constants. *J.Chem. Educ.* **2008,** *85* (4), 532.
